# A clinical trial to evaluate the effect of statin use on lowering aldosterone levels

**DOI:** 10.1186/s12902-020-00587-4

**Published:** 2020-07-14

**Authors:** Ezra S. Hornik, Anne E. Altman-Merino, Andrew W. Koefoed, Kayla M. Meyer, Isabella B. Stone, Jessica A. Green, Gordon H. Williams, Gail K. Adler, Jonathan S. Williams

**Affiliations:** Division of Endocrinology, Diabetes, and Hypertension, Brigham and Women’s Hospital and Harvard Medical School, 221 Longwood Ave, Boston, MA 02115 USA

**Keywords:** Aldosterone, Statins, Cardiovascular health, Metabolic health, Hypertension, Hyperlipidemia, Mineralocorticoid, Simvastatin, Pravastatin

## Abstract

**Background:**

Statins are the first-line pharmaceutical agent in the management of hypercholesterolemia and cardiovascular (CV) risk reduction, and the most commonly prescribed class of drugs worldwide. Studies describing CV risk reduction independent of LDL-cholesterol lowering have evoked an interest in the pleiotropic mechanisms of statins’ benefits. We recently demonstrated that administration of statins in animal models lowers aldosterone levels and observed an association between statin use and reduced aldosterone levels in two human cohorts, with lipophilic statins displaying a greater effect than hydrophilic statins. Therefore, we designed a randomized, placebo-controlled, double-blinded intervention study to assess whether statin treatment lowers aldosterone in a type-dependent manner in humans, with simvastatin (lipophilic) showing a greater effect than pravastatin (hydrophilic).

**Methods/design:**

One hundred five healthy participants will be recruited from the general population to enroll in a 12-week, randomized, placebo-controlled, double-blinded, 3-arm clinical trial. Ninety participants are anticipated to complete the protocol. After baseline assessment of aldosterone levels, participants will be randomized to daily simvastatin, pravastatin, or placebo. Aldosterone levels will be assessed after 2 days on study drug and again after 6 weeks and 12 weeks on study drug. Prior to each aldosterone assessment, participants will consume an isocaloric sodium and potassium-controlled run-in diet for 5 days. Assessments will occur on an inpatient research unit to control for diurnal, fasting, and posture conditions. The primary outcome will compare 12-week angiotensin II-stimulated serum aldosterone by study drug. Secondary outcomes will compare baseline and 12-week 24-h urine aldosterone by study drug.

**Discussion:**

Results from this rigorous study design should provide strong support that statins lower aldosterone levels in humans. These results may explain some of the beneficial effects of statins that are not attributed to the LDL-lowering effect of this important class of medications. Results would demonstrate that statin lipophilicity is an important attribute in lowering aldosterone levels. The outcomes of this program will have implications for the design of studies involving statin medications, as well as for the differential use of classes of statins.

**Trial registration:**

ClinicalTrials.gov; NCT02871687; First Posted August 18, 2016.

## Background

Cardiovascular disease (CVD) is the leading cause of mortality in the United States, and as such, CVD and reduction of its risk factors are important and widespread subjects for medical research [1]. Hypercholesterolemia is a major risk factor for CVD, and as the established first-line treatment for it, statins are the most commonly prescribed cholesterol-lowering medications in the U.S. [2]. Randomized trials have robustly demonstrated the benefit of statin therapy to reduce CVD, including coronary events, heart failure, and ischemic stroke [2–5]. While this benefit has been primarily attributed to the LDL-lowering effect of statins, subsequent studies suggest that the benefits of statin therapy may arise from pleiotropic actions distinct from the effect on lipid levels [6–8].

**Fig. 1:**
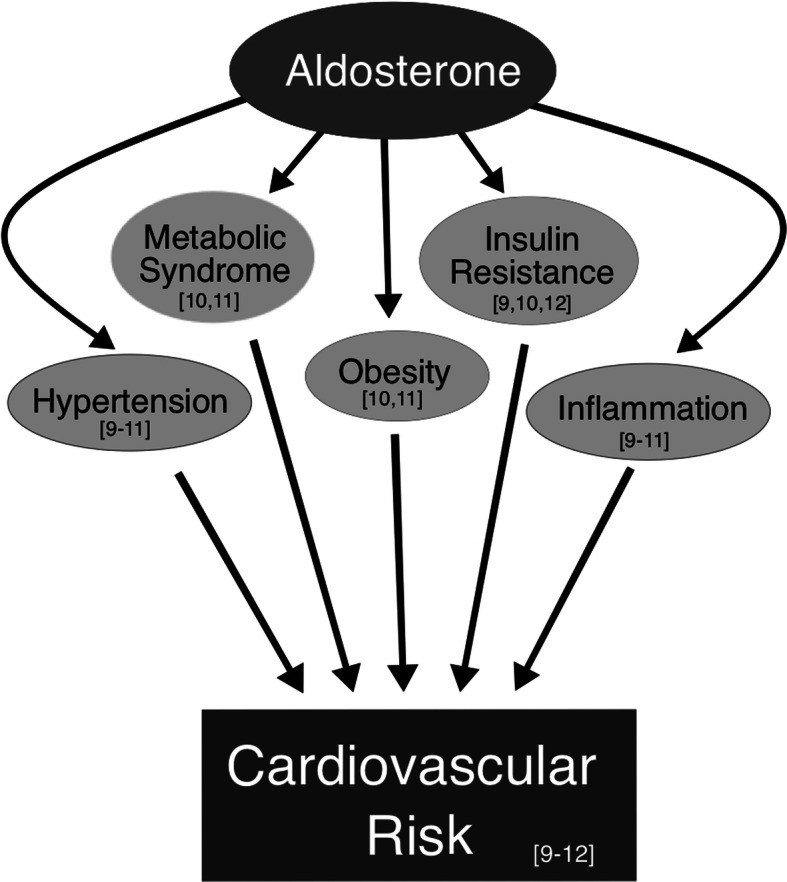
Aldosterone and its effects on cardiovascular risk factors. In addition to its primary function as a blood pressure and volume regulator, aldosterone influences several factors related to cardiovascular risk; insulin resistance [9, 10, 12], hypertension [9–11], and obesity [10, 11]. Additionally, increased aldosterone secretion is associated with metabolic syndrome [10, 11] and higher degrees of inflammation in the kidneys, liver, vasculature, and systemically [9–11]. Thus, aldosterone excess or excess mineralocorticoid activity exacerbates cardiovascular risk and mitigation should be of benefit

Previous research by our group and others has associated increased aldosterone levels with more severe hypertension, insulin resistance, reduced beta cell function, and exacerbated CVD, as well as higher incidence of metabolic syndrome and CV injury [9–12]. Thus, elevated aldosterone is considered a CV risk factor (Fig. 1).

Accordingly, we investigated whether statin use was associated with aldosterone levels using a well-established international dataset (HyperPATH) designed to meticulously measure aldosterone in normotensive, hypertensive, and diabetic participants [13]. We observed lower aldosterone levels in hypertensive participants who self-reported at least 3 months of statin use versus those who reported no statin use. These results were replicated in a separate cohort of diabetic participants under the same study conditions [13]. Furthermore, this effect appeared to be dependent on the type of statin; the extent of aldosterone lowering was more significant with lipophilic statins than with hydrophilic statins.

To further illustrate the relationship between statins and aldosterone secretion, ex vivo studies were conducted in rodent adrenals, in which isolated zona glomerulosa (ZG) cells were preincubated with statins of varying lipophilicity [13]. Following incubation with angiotensin II, a known secretagogue of aldosterone, ZG cells exposed to a statin demonstrated significantly lower aldosterone levels than those that had not been preincubated, with lipophilic statins showing greater aldosterone-lowering than hydrophilic statins. This statin effect was absent in corticosterone-secreting adrenal zona fasciculata cells, further supporting the hypothesis of a specific aldosterone-lowering action of statins.

In light of these findings, we hypothesize that in humans, treatment with statins lowers aldosterone levels, and in turn, could explain the beneficial pleiotropy of this class of medications with regard to non-LDL-cholesterol CV risk reduction. Thus, we propose a prospective, randomized, double-blinded, placebo-controlled trial in humans to assess whether treatment with statins results in lower aldosterone levels (Aim 1), and whether the effect of statin treatment is dependent on the type of statin, with simvastatin (lipophilic statin) hypothesized to have a greater aldosterone-lowering effect than pravastatin (hydrophilic statin) (Aim 2).

## Methods/design

### Study population and recruitment

This study will enroll 105 healthy adults, ages 18–70 years, with the intent to have data from 90 participants available for analysis. Participants will be recruited from the general population of the greater Boston area. Eligibility will be assessed at in-person screening visits involving laboratory blood and urine assessment, measurement of vital signs, electrocardiogram, physical examination, and a detailed medical history.

Inclusion criteria will require blood pressure < 140/90 mmHg and > 90/50 mmHg, body mass index 19–40 kg/m^2^, normal screening laboratory values for serum sodium, potassium, glucose, liver enzymes, estimated glomerular filtration rate (> 60 mL/min/1.73m^2^), HbA1c%, thyroid stimulating hormone (TSH), and hemoglobin, normal 12-lead electrocardiogram, and a negative pregnancy testing for women of childbearing potential. Participants with stable thyroid disease may be included if TSH is normal and there have been no changes in thyroid medication for > 6 months.

Exclusion criteria include abnormal TSH, triglycerides > 500 mg/dl, LDL < 70 and > 200 mg/dl, any prescription medication except thyroid medications, hormonal methods of birth control, pregnancy or current breastfeeding, alcohol intake > 12 oz. per week, tobacco or recreational drug use, any prior use of statin therapy, history of coronary disease, diabetes, hypertension, stroke, kidney disease, psychiatric illness, malignancy, preeclampsia, or illness requiring overnight hospitalization in the past 6 months. All participants will provide written informed consent after review with a study physician. All study procedures are approved by the Partners (now Mass General Brigham) Institutional Review Board. The study was registered with ClinicalTrials.gov (NCT02871687).

### Study protocol overview

**Fig. 2:**
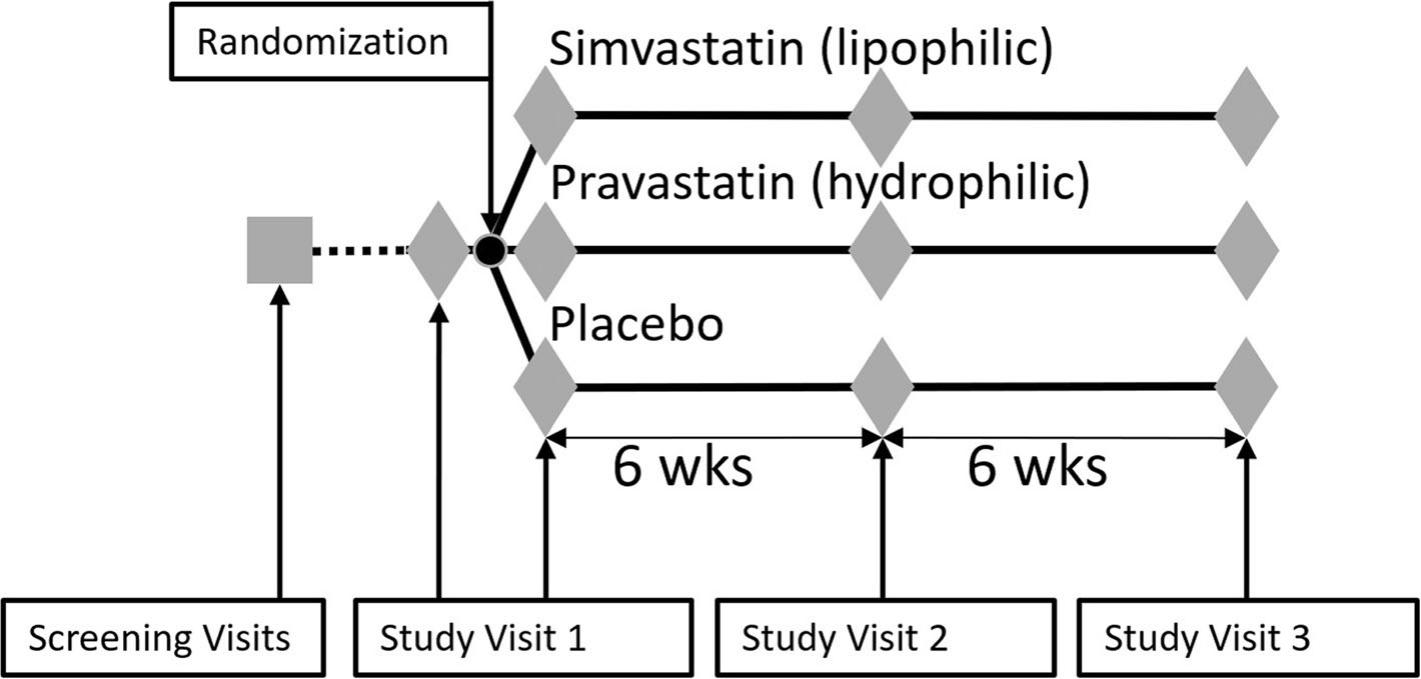
Study schema. Participants will be enrolled in the study after completing screening visits. At Study Visit 1, aldosterone levels before and after angiotensin II stimulation will be assessed at baseline before participants are randomized to either simvastatin 20 mg, pravastatin 40 mg, or placebo. Aldosterone secretion will then be assessed 1 day after initiation of study drug in order to determine the acute effects of statin use. Aldosterone secretion will be assessed after 6 weeks at Study Visit 2 and after 12 weeks at Study Visit 3 in order to determine the chronic effects of statin use. Approximately 5 days before Study Visit 2, participants will have their lipid levels checked. If their LDL-cholesterol has decreased by less than 35% since screening, study drug dose will be doubled for the final 6 weeks of the study starting after Study Visit 2. A 5-day low-sodium diet will precede each study assessment. The primary endpoint is comparison of 12-week angiotensin II-stimulated aldosterone secretion between the simvastatin and placebo groups

A detailed study schema is shown in Fig. 2. All study visits will occur in the Inpatient Laboratory of the Center for Clinical Investigation (CCI) at Brigham and Women’s Hospital, Boston, MA in order to stabilize renin-angiotensin aldosterone system (RAAS) activity by controlling posture, time of day, and dietary intake. Each participant will complete three inpatient study visits for RAAS assessment: a baseline visit (Study Visit 1), 6-week visit (Study Visit 2), and 12-week visit (Study Visit 3). Prior to each study visit, they will consume a controlled diet for 5 days. A 24-h urine collection starting the morning of day 5 will ensure compliance with the diet prior to inpatient study procedures. Baseline RAAS assessment will occur during Study Visit 1 prior to drug administration, along with reassessment after the participant receives 2 doses of study drug to evaluate the acute effect of statins on RAAS activity. Study Visits 2 and 3 will occur after 6 and 12 weeks on the study drug, providing two time points from which to measure the chronic effect of statin on RAAS activity.

#### Low-sodium study diet

All study assessments (Study Visits 1, 2, and 3) will be conducted after participants have consumed a controlled diet for 5 days. This diet will have low sodium content to maximally stimulate the RAAS and enhance signal detection similarly in all participants. This run-in diet is calculated, prepared, and provided by the BWH CCI Nutrition and Metabolic Research Core and includes daily breakfast, lunch, dinner, and snacks. The diet is isocaloric and contains 10 mEq sodium, 100 mEq potassium, and 1000 mg calcium per day.

#### Randomization of study drug

Prior to Study Visit 1, participants will be randomized to simvastatin 20 mg per day, pravastatin 40 mg per day, or placebo using a block randomization schedule with block size of 3 and maintaining equal distribution of female/male sex. Double-blinding will be maintained by use of the Investigational Drug Services Pharmacy at BWH who will prepare and dispense study drug and assign randomization.

### Baseline assessment and acute effect of statins on aldosterone secretion (study visit 1)

After 5 days on the run-in controlled diet, participants will complete a Posture Study after fasting overnight. The Posture Study is designed to further stimulate the RAAS by requiring that participants remain in an upright (standing) posture for at least 45 min. During this time, blood will be drawn for measurement of RAAS components. Timing of the Posture Study is restricted to between 8:00 AM and 10:00 AM to reduce diurnal variability in our outcomes.

Participants are then admitted to the Inpatient Facility of the CCI for a 2-night stay. Upon admission on Day 1, sodium concentration will be measured from an aliquot of the ongoing 24-h urine collection to ensure compliance with the study diet as previously described. After midnight, participants will remain supine, including for voiding and defecation, without head elevation and will remain fasting until the study procedures are completed the next morning.

Two intravenous lines (IV) will be placed (right forearm and left forearm) on the morning of Day 2 at least 2 h prior to study assessments. One IV will be used for serial blood draws to avoid stimulation from repeat needle sticks. The second IV will be used for administration of angiotensin II (Giapreza, La Jolla Pharmaceutical, San Diego, CA) to stimulate aldosterone secretion. One hour after IV insertion, blood will be withdrawn for baseline assessment of renin (plasma renin activity), aldosterone, cortisol, and angiotensin II. Following the initial assessment, blood will be drawn for assessment of the same hormones after a graded, weight-based infusion of angiotensin II. The first dose of angiotensin II (1 ng/kg/min) is typically sub-pressor. After 30 min of infusion at this dose, blood will be drawn for hormone assessment and the angiotensin II dose will be increased to 3 ng/kg/min to create a mild pressor effect. After 30 min of infusion at this increased dose, the final blood samples will be drawn for hormone assessment and the angiotensin II infusion will be discontinued. After completing the angiotensin II infusion, participants will receive their first dose of assigned study drug. Urine collections will continue throughout the day and will be assayed for electrolytes and aldosterone upon completion. Participants will continue the low-sodium diet until approximately midnight on their second night in the CCI, at which point they will remain fasting and supine.

On the morning of Day 3 at 6:00 AM, participants will receive a second dose of study drug. Baseline and angiotensin II study procedures that were conducted on Day 2 will be repeated. Upon completion of study procedures on Day 3, participants will be discharged with instructions to continue taking the assigned study drug each evening.

*Anticipated Results from Study Visit 1: Two doses of statin drug will decrease angiotensin II-stimulated aldosterone secretion relative to placebo. Simvastatin treatment will result in a larger blunting of angiotensin II-stimulated aldosterone secretion compared to pravastatin.*

### Chronic effect of statins on aldosterone secretion (study visits 2 & 3)

### Study visit 2

After approximately 6 weeks of daily study drug, participants will begin the same 5-day study diet run-in prior to their second inpatient study visit. They will then be admitted to the CCI for one night and repeat the baseline and angiotensin II infusion procedures described during Study Visit 1.

*Anticipated Results from Study Visit 2: After 6 weeks, statin drugs will decrease angiotensin II-stimulated aldosterone secretion relative to placebo, and simvastatin treatment will result in a larger blunting of angiotensin II-stimulated aldosterone secretion compared to pravastatin.*

Study Drug dose escalation: Blood will be drawn to assess LDL-cholesterol level after approximately 6 weeks on study drug (approximately 5 days before Study Visit 2). Following completion of Study Visit 2, the dose of study medication will be doubled (40 mg simvastatin, 80 mg pravastatin, 0 mg placebo) if the LDL-cholesterol has decreased less than 35% compared to its level at screening. If the LDL-cholesterol level has decreased more than 35% since screening, they will remain on the same dose. Participants will be unaware of LDL-cholesterol level or dose changes to ensure blinding. Participants will then continue their daily study medication for an additional 6 weeks after completion of Study Visit 2.

### Study visit 3

After approximately 12 weeks of treatment with study drug, participants will complete the same controlled diet run-in for 5 days followed by admission to the CCI for one night. They will repeat all study procedures as in Study Visit 2. After completing Study Visit 3, participants will have finished study participation and will discontinue the study medication. LDL-cholesterol values from the screening visit will then be disclosed with recommended healthcare provider follow-up if indicated.

*Anticipated Results from Study Visit 3: After 12-weeks, statin drug will decrease angiotensin II-stimulated aldosterone secretion relative to placebo, and simvastatin treatment will result in a larger blunting of angiotensin II-stimulated aldosterone secretion compared to pravastatin.*

### Safety and monitoring

#### Blood pressure

During the angiotensin II infusion, blood pressure will be measured every 2 min by an automated sphygmomanometer (Dinamap, General Electric Healthcare, Chicago IL) and monitored throughout the duration of the infusion by a trained research observer. IV infusion of angiotensin II is expected to increase blood pressure. If during the infusion the diastolic blood pressure rises above 105 mmHg or the systolic blood pressure rises above 180 mmHg on three consecutive readings, verified by a manual cuff reading, the infusion will be terminated. If participants develop symptoms such as headache, chest pain, palpitations, or nausea, the infusion will be terminated, and appropriate medical attention provided. The use of angiotensin II in this protocol will be identical to previous protocols [13–15] and has been performed safely and without event in over 1250 participants. Blood pressure measurements will be collected for 10 min post-infusion to ensure a return to baseline blood pressure, and the principal investigator will review all collected blood pressure measurements.

#### Study drugs: simvastatin and pravastatin

Statin drugs (simvastatin and pravastatin) will be used in this study. They may be used “off label” in that some individuals without hypercholesterolemia or indication for statin therapy will receive these medications. To reduce the risk of side effects, all participants will be screened for predisposing conditions prior to enrollment, and participants will be contacted weekly to assess for development of symptoms of drug-related side effects, including myopathy, transaminitis, rhabdomyolysis, renal failure, upper respiratory infection, headache, abdominal pain, constipation, and nausea. If symptoms suggest possible side effects, an additional study visit, including lab work, may be required to evaluate further. Depending on this evaluation, patients may be discontinued from the study and appropriate clinical follow-up arranged.

#### Laboratory results

All laboratory studies are reviewed by a study physician. Clinically significant abnormalities will either result in a redraw or immediate exclusion. Clinically significant abnormalities will be discussed with the participant by a study physician who will advise follow-up.

#### Data safety monitoring plan

Data and safety will be monitored during a monthly research group meeting. The Cardiovascular Endocrinology Research Group includes more than 5 patient-oriented clinical investigators who are all endocrinologists with detailed knowledge of human participants research and the medications used in this study. At each meeting, study staff present recruitment status, adverse events or safety issues, protocol deviations, and other pertinent study-related issues to the group. The Cardiovascular Endocrinology Research Group reviews each adverse event and formulates a plan to address it.

### Laboratory measurements

Twenty-four hour urines for sodium, potassium, aldosterone, and creatinine will be collected on each day of the inpatient visits. Sodium and potassium will be measured by flame photometry (Cole-Parmer, Vernon Hill, IL, USA). Aldosterone will be measured using an assay from Immuno-Biological Laboratories Inc., Minneapolis, MN, USA. Serum aldosterone and plasma renin activity (Immuno-Biological Laboratories Inc., Minneapolis, MN, USA) and serum cortisol (Beckman Coulter, Brea, CA, USA) will be measured from samples drawn at three time points (before infusion, after 1 ng/kg/min infusion, and after 3 ng/kg/min infusion) during inpatient Visits 1, 2, and 3, and also during the Posture Visit. A lipid panel will be taken at the lipid check visit.

### Statistical analysis

Sample size calculations were derived from our observational report [13] that described the relationship between statin users and circulating aldosterone levels. In this study, the measured aldosterone level in response to angiotensin II infusion (3 ng/kg/min for 45 mins) within each participant group (self-reported statin users and non-statin users) was normally distributed with a mean population standard deviation of 16 ng/dL. If the true difference between an active study drug (simvastatin or pravastatin) and placebo mean aldosterone level is 14 ng/dL (based on the population means from our observational study), we will need to study 30 individuals in each of the three study arms to be able to reject the null hypothesis that the aldosterone levels across arms is equal with probability (power) 0.85. The Type 1 error probability associated with this test is 0.025, since 2 study drugs will be compared independently with placebo. Secondary endpoints will report the difference between simvastatin and pravastatin and evaluate the dose-relationship and that with LDL-cholesterol lowering effects by drug. We will aim to enroll 105 individuals and anticipate a 15% drop-out rate to enable evaluation of 90 individuals (30 per arm).

The three arms of the study population will be compared for baseline characteristics to assess quality of randomization assignment based on age, BMI, sex, race, and aldosterone levels. We anticipate that compared to placebo, the aldosterone response to angiotensin II after 12 weeks of simvastatin will be significantly muted, indicating a decrease in aldosterone secretory activity. Based on our previous report in isolated adrenal ZG cells, we anticipate that the less lipophilic statin (pravastatin) will have little ability to dampen the aldosterone response to angiotensin II infusion and will be similar to placebo.

## Discussion

This study is the first double-blinded, randomized, placebo-controlled trial in humans designed to elucidate the effect of statin use on aldosterone secretion. The fundamental premise is based on the convergence of two well-established scientific facts: 1) dysregulated aldosterone activity is linked to increased CV risk [9–12], and 2) statin medications demonstrate pleiotropy in CV risk reduction [6, 16, 17]. Observational reports from two study populations provide the epidemiological support to justify the need for a clinical trial. We previously reported in a general normotensive and hypertensive population that users of statin drugs displayed lower circulating aldosterone levels than non-users. This was replicated in a general population of individuals with Type 2 diabetes mellitus [13]. Further, adrenal ZG cells from mice secreted less aldosterone when incubated with a statin. Interestingly, when exploring the degree to which aldosterone secretion was decreased, both observations in the human cohorts and incubated adrenal cells revealed a greater effect with increasing statin lipophilicity. Thus, results from this study could have substantial impact in clarifying the non-LDL-cholesterol lowering benefit of statin drugs on CV risk reduction, which types of statins might have preferential benefit, and which populations may receive particular CV protection.

**Fig. 3:**
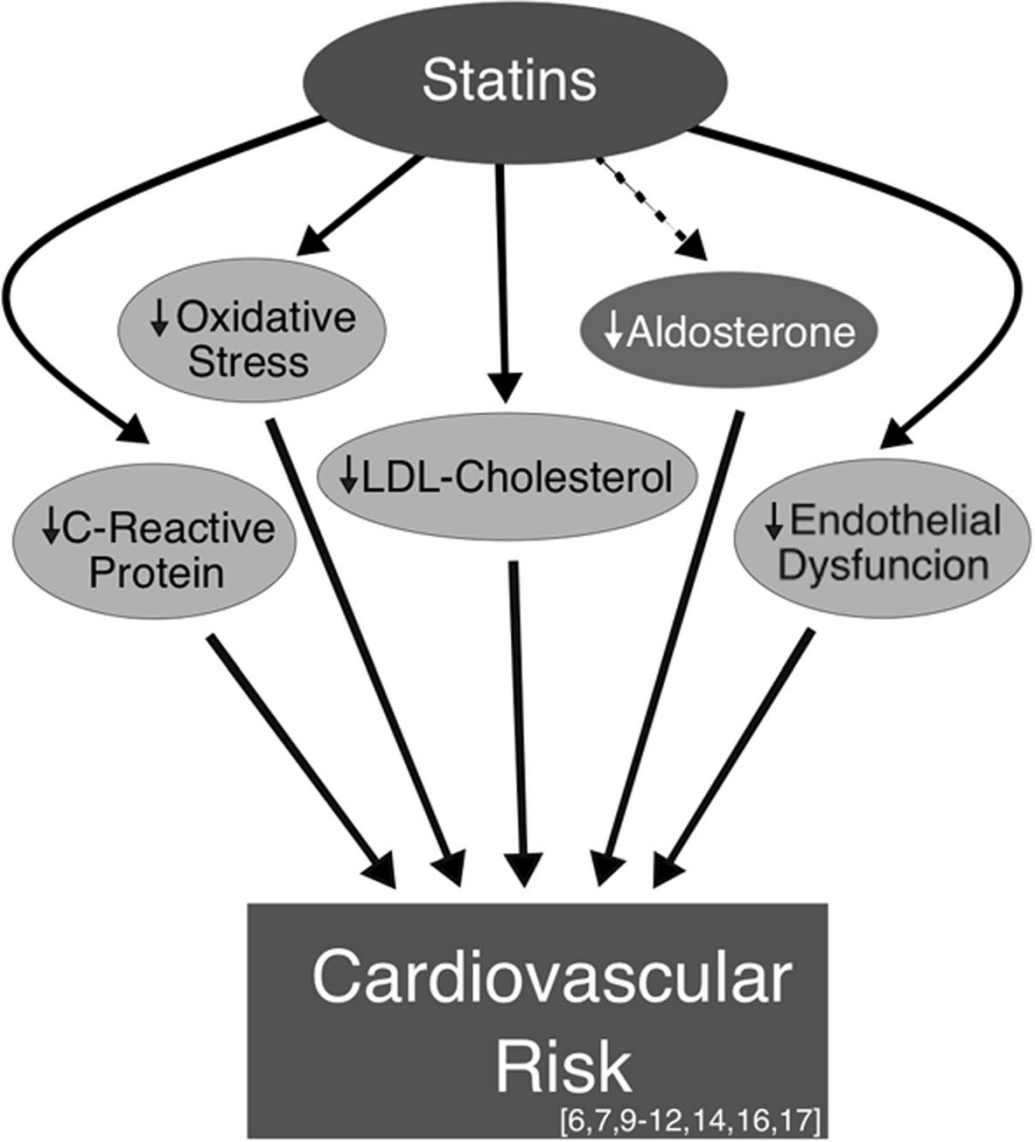
Beneficial pleiotropic effects of statin medications. Data from the past decade have shown that in addition to their classic LDL-cholesterol lowering effects, statins also function to improve cardiovascular health by mitigating oxidative stress, improving endothelial dysfunction through increased NO function, and decreasing high-sensitivity C-Reactive Protein levels. We propose that statins’ aldosterone-lowering effect represents another mechanism by which statins reduce cardiovascular risk apart from their influence on LDL-cholesterol

Currently, the beneficial effects of statins on aldosterone levels are generally assumed to be secondary to their LDL-cholesterol lowering effects. However, there is evidence that in addition to inhibition of HMG-CoA reductase to lower LDL-cholesterol, statins also reduce oxidative stress [16], endothelial dysfunction [6, 16], and C-Reactive Protein [16, 17]. This study seeks to elucidate an additional pleiotropic effect of statins by demonstrating their aldosterone-lowering effects and thus their impact on CVD (Fig. 3). If our hypothesis proves to be true, it will substantially change how we approach enhancing statins’ beneficial effects from a singular one of reducing lipid levels to a dual approach of lowering aldosterone and LDL-cholesterol. These results could provide an answer to why some statins produce a beneficial CV effect when LDL-cholesterol levels are already normal or low, and why some statins that reduce LDL-cholesterol levels less than other statins have greater protective effects.

Finally, our results will likely shift the focus of new drug development away from molecules that more effectively lower only LDL-cholesterol, and towards drugs that are better optimized to lower both LDL-cholesterol and aldosterone.

We have taken careful consideration in designing this study to reduce factors known to affect RAAS activity in order to enhance signal detection and reduce confounding. These considerations are based on decades of experience in conducting dozens of studies related to RAAS manipulation and assessment. First, we chose to enroll individuals from a general population who did not have evidence of overt cardiometabolic derangements to eliminate the influence of medication use or complex medication washout programs. Second, we will use a standardized and controlled diet prior to each hormonal assessment. Third, all participants will be studied in an inpatient clinical research center which enables control over feeding and fasting, diurnal variation, body posture, and standardized operating procedures to minimize environmental impact on hormonal variation.

In order to increase the ability to detect a change in aldosterone levels across study arms, we have included several factors to stimulate aldosterone secretion, including a standardized low-sodium and controlled potassium diet run-in period prior to each RAAS assessment. In addition to stimulating aldosterone secretion, this diet will provide a uniform baseline within and across participant assessment throughout the trial. We include a 24-h urine collection at the end of each diet run-in and will only permit assessment if sodium values are below 30 mmol/day. Finally, we include a graded angiotensin II infusion (a primary stimulator of aldosterone secretion) across a range that we have used in thousands of similar participants over the last 30 years [13–15].

To explore the relationship between lipophilicity and effect on aldosterone secretion, we have included two active drug comparator arms in addition to a placebo arm. Our statistical design enables comparison of both drugs independently against placebo, but also affords the ability to gauge across-statin drug impact on aldosterone reduction. In order to maximize the effect of statins to lower aldosterone, we included an intermediate 6-week assessment followed by a study drug dose escalation based on LDL-lowering. If the 6-week LDL-cholesterol level is not reduced by at least 35% from baseline, then the dose of study drug is doubled, allowing an additional 6 weeks at a higher dose in those individuals before final assessment at week 12.

There are several limitations in the study design which may impact the final results. These include standard risks related to study recruitment, retention, and adherence. As mentioned previously, these risks are greatly mitigated by the study design and decades of experience with similar trials. Several assumptions could also influence the study outcome. As opposed to the observational reports in hypertensive and diabetic populations, we chose to study a relatively healthy population in order to reduce confounding and complexity. Therefore, the study population may represent a demographic for whom CV risk, and therefore aldosterone production, is not an immediate concern. However, we strongly believe that these results will be generalizable to disease state populations, and also that aldosterone production in healthy individuals is pertinent due to the chronic nature of CVD and the accumulation of its risk factors over a lifetime. We have attempted to overcome this limitation by instituting several measures to enhance signal detection for aldosterone and reduce environmental noise. Our primary outcome is based on the change in aldosterone levels in response to angiotensin II infusion across study arms. However, this study design includes evaluation after 1 day and 6 weeks on a statin, in addition to the 12-week time-point at which the primary endpoints are evaluated. These additional time-points will lend a preliminary understanding of the time frame by which statins influence aldosterone secretion - namely, whether the effect can be seen acutely after 1 day and/or after 12-week statin treatment.

Data supporting and identifying synergistic benefits of the multiple effects of the widely used statin class of medications will likely have significant clinical implications on the future management of hypertension and other CVD. This is of particular importance if individual statin chemical moieties impart preferential benefit, which could fuel development of agents that reduce both cholesterol and aldosterone levels.

## Data Availability

Not applicable.

## Abbreviations

CV: Cardiovascular
CVD: Cardiovascular Disease
ZG: Zona Glomerulosa
TSH: Thyroid Stimulating Hormone
CCI: Center for Clinical Investigation
IV: Intravenous
RAAS: Renin-angiotensin aldosterone system
